# MicroRNA调控肿瘤耐药的研究进展

**DOI:** 10.3779/j.issn.1009-3419.2014.10.06

**Published:** 2014-10-20

**Affiliations:** 300052 天津，天津医科大学总医院，天津市肺癌研究所，天津市肺癌转移与肿瘤微环境重点实验室 Tianjin Key Laboratory of Lung Cancer Metastasis and Tumor Microenvironment, Tianjin Lung Cancer Institute, Tianjin Medical University General Hospital, Tianjin 300052, China

**Keywords:** MicroRNA, 肿瘤耐药, MDR, 靶点, MicroRNA, Chemo-resistance, MDR, Targets

## Abstract

化学药物治疗（简称化疗）是目前治疗恶性肿瘤的主要手段之一，但肿瘤的多药耐药（multidurg resistance, MDR）是临床化疗失败的主要原因之一，然而肿瘤的耐药机制至今尚未被完全阐明。microRNA是近年来生命科学新的研究热点之一，microRNA通过在转录后水平调控基因表达而参与调控一系列的生命活动，包括细胞增殖、凋亡、脂肪代谢、神经元发育、激素分泌、肿瘤血管生成、干细胞分化、肿瘤细胞浸润及转移等多种生理和病理过程。最近的研究表明，microRNA对多基因表达的调控具有高效性和特异性，对靶基因的异常调控可能构成肿瘤耐药机制，是肿瘤耐药复杂性调控的重要构成部分。因此，对microRNA与肿瘤耐药性的研究具有现实意义。本文对近年来肿瘤耐药性的研究，microRNA调控肿瘤耐药机制及microRNA作为肿瘤耐药治疗潜在靶点作一综述。

## Abstract (en)

Chemotherapy is one of the primary treatment for malignant tumors. Tumor multidrug resistance (MDR) is a major cause of clinical failure of chemotherapy; however the mechanisms of chemo-resistance have not been fully elucidated. Recently, microRNA is one of the new hotspots in life science. MicroRNA regulates the expression of genes and plays roles a series of life events by post-transcriptional regulations, including cell proliferation, apoptosis, fat metabolism, nervous development, hormone secretion, tumor vessels generation, stem cell differentiation, tumor cell invasion and metastasis, and other physiological and pathological processes. Recent studies show that microRNA regulates the expression of multiple genes with high efficiency and specificity. The abnormal regulation of target genes by microRNA is responsible for tumor chemo-resistance, this may be an important component of the complexity of the regulation of chemo-resistance. Therefore, the study of microRNA and tumor drug resistance has profound practical significance. In this review, recent studies of tumor drug resistance, regulation of tumor drug resistance by microRNA, and microRNA as a potential target for tumor drug resistance therapy are reviewed.

## 1 肿瘤细胞的耐药性

恶性肿瘤是严重威胁人类健康的常见病及多发病，1943年，耶鲁大学的Gilman等^[1]^首先将氮芥应用于淋巴瘤的治疗，揭开了现代肿瘤化疗的序幕。随后新的抗肿瘤药物不断出现，化疗成为肿瘤主要的治疗方法之一，但是如同细菌易对抗生素产生耐药性一样，肿瘤细胞也常对化疗药物产生耐药性，而且更为普遍，成为导致化学治疗失败的主要原因之一。因此肿瘤耐药是肿瘤治疗急需解决的关键问题之一。

肿瘤细胞群体具有内在的、高度有序发展的抗药能力，无论是细胞毒类药物还是靶向药物，均未能克服耐药问题。肿瘤的耐药性是影响化疗疗效和肿瘤根治的主要原因，至今仅有少数组织来源的晚期肿瘤，可以通过药物治愈，而且即使是这些肿瘤，也可能复发并发生耐药。肿瘤细胞的耐药性包括原发性耐药（intrinsic resistance）和获得性耐药（acquired resistance）^[2]^。原发性耐药亦称天然性耐药、内在性耐药，指肿瘤的耐药性是天然的，在肿瘤细胞用药之前就已经存在，化学治疗对这些肿瘤的疗效较差；多数肿瘤细胞的耐药性属于获得性耐药亦称继发性耐药，是在化学治疗过程中化疗药物诱导逐渐产生的。肿瘤细胞一旦产生耐药性，化疗药物就不能发挥完全的抗癌作用杀死癌细胞，即使大多数的肿瘤被杀死，而这一小部分具有抗药性的癌细胞依然会继续生长，造成癌症复发。进而，化学治疗疗效越来越差，这是导致恶性肿瘤最终无药可用的重要原因之一。此外，根据肿瘤的耐药谱还可以分为原药耐药（primary drug resistance）和多药耐药（multidrug resistance, MDR）。前者是指肿瘤细胞只对开始接触的原药不敏感，而后者则除了对开始诱导的药物有抗性之外，对其他未接触过而且结构不同、作用机制各异的药物也产生交叉耐药性。这个概念是1970年最先由Biedler和Riehm提出的^[3]^。肿瘤的耐药机制非常复杂，肿瘤的MDR与多药耐药基因（multi-drug resistant genes）过表达密切相关，最近的研究^[4]^发现，化疗药物以外的损伤因素也可以使多药耐药基因表达增强，而这些损伤因素所引起的多药耐药基因的高表达均可以使细胞对后来的其他损伤因素产生耐受，因此，多药耐药基因应改称耐多种损伤基因似乎更能体现其内涵。迄今为止，肿瘤的多药耐药是肿瘤治疗尚未解决的难题之一。

## 2 microRNA的命名、生物合成和功能

微小RNA（microRNA, miRNA）是长度约22个核苷酸（nucleotide, nt）的单链小RNA分子，其中21 nt-23 nt长度的miRNA占大多数，约为84%（图 1）^[5]^。属于非编码RNA（non-protein-coding RNAs, ncRNAs）的一种。非编码RNA是基因组转录产物中非蛋白编码的部分，曾被认为是转录“噪声”，主要包括miRNA、小干扰RNA（small interfering RNAs, siRNAs）、piwi蛋白相互作用RNA（PIWI-interacting RNAs, piRNAs）和长链非编码RNA（long ncRNAs, lncRNAs）。目前，这些最初被认为是转录副产物的物质被发现在生物信息流由DNA模板向蛋白效应分子转化过程的多个层面发挥着不同形式的调节作用。最近的研究表明，miRNA对多基因表达的调控具有高效性和特异性，对靶基因的异常调控可能构成肿瘤耐药机制，是肿瘤耐药复杂性调控的重要构成部分。这些发现改变了“RNA仅仅是DNA和蛋白之间媒介”的传统观念。

**1:**
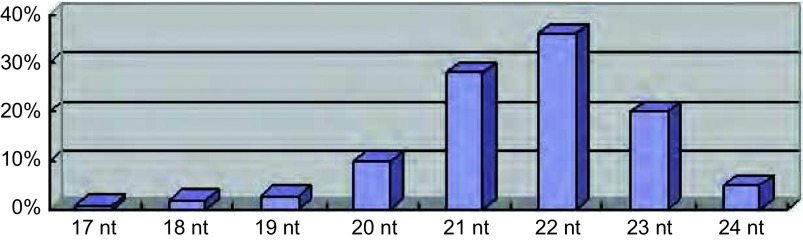
已知的microRNA核苷酸长度的分布情况 The distribution of the length of known microRNA nucleotides

### 2.1 MiRNA的命名

miRNA是目前ncRNA中被研究的最多的成员，到目前为止，人们已在植物、线虫、果蝇，动物中发现了一万五千多个miRNA，其中人类细胞中已发现的有1, 048个^[6]^。miRNA的命名原则：miRNA可简写成miR，再根据其被克隆的先后顺序加上阿拉伯数字，如miR-22。一般而言，数字越小，发现越早；高度同源的miRNA在数字后加上英文小写字母（a、b、c••••••），如miR-181a、miR-181b、miR-181c；位于基因组不同位点但具有相同成熟体序列的miRNA，应在序列后添加短线和阿拉伯数字以示区别，如miRNA-34a-1、miRNA-34a-2；miRNA前体经过一个发夹环结构，具备两个臂，但通常只有一个会被选择成为最终的miRNA，那么被降解掉的另外一条臂则以“^*^”表示；物种缩写应置于miRNA之前，如has、mmu和rno分别代表人、小鼠和大鼠；确定命名规则之前发现的miRNA应保留原来名字，如lin-4^[7]^。

### 2.2 MiRNA的生物合成

MiRNA可能来自于基因组的基因间隔区或者pre-mRNA的内含子中，但是不论其来自何处，其加工成熟机制都是相同的。首先，在细胞核中，RNA酶Ⅲ Drosha-DGCR复合体在miRNA原初转录物（pri-mRNA）基部切割双链形成60 nt-70 nt的5’末端具有磷酸基，3’末端具有二核苷酸突出（over hang）的茎环中间体---前体miRNA（pre-miRNA）。然后，转运蛋白Exportin-5通过识别pre-miRNA的3’端的二核苷酸突出信号而与pre-miRNA结合，依赖Ran-GTP将pre-miRNA输出到细胞质。植物中是由Exportin-5的同源物HASTY负责miRNA前体的输出^[8]^。最后，Dicer识别pre-miRNA双链的5’末端磷酸及3’末端突出，在距茎环大约两个螺旋转角处切断螺旋体的双链，产生一个结构类似于siRNA的二聚体，其中成熟的miRNA来自于pre-miRNA的一条臂，pre-miRNA的另外一条臂产生一个长度与miRNA相同的片段，即miRNA^*^^[9]^。在经过切割和核质转运后，RNA解旋酶作用于miRNA:miRNA^*^二聚体，其中miRNA进入RNA诱导的沉默复合物（RNA-induced silencing complex, RISC）中发挥作用，miRNA^*^则被降解^[10, 11]^。

### 2.3 MiRNA的生物学功能

MicroRNA是通过与靶mRNA的3’-UTR碱基配对的方式来执行对靶mRNA的转录翻译抑制的功能。在动物中，microRNA与靶mRNA进行不完全配对执行转录后翻译抑制的作用。但是如果动物miRNA与靶序列完全互补，则进入RNA干扰路径指导靶序列降解。在筛选果蝇生长缺陷突变株的过程中发现了Bantam基因座。该基因座编码了果蝇幼虫抑制程序性死亡和促进细胞增殖的miRNA。Bantam miRNA大量表达并抑制Hid mRNA的翻译。Hid是程序性死亡的主要激活因子。Bantam类似于线虫的miR-80/82，这表明miR-80家族能控制线虫的细胞死亡和增殖^[12]^。动物中的miRNA还能影响细胞分化。miR-23与*Hes1*基因具有较完全的互补性（大约77%）。Hes1是一种基本的螺旋-环-螺旋结构，它只在未分化细胞中表达。将人工合成的miR-23加到未分化的NT2神经细胞中，可以发现细胞内的Hes1水平大幅下降。但是将人工合成的miR-23突变体加入到未分化NT2细胞中，Hes1水平保持不变，且其含量与将野生型miR-23加入到已分化的NT2细胞中是一样的。这表明miR-23在翻译水平上抑制*Hes1*基因的表达，影响细胞分化^[13]^。在植物中，由于miRNA与mRNA互补程度高，故可用来预测植物microRNA的靶位点。实验证据^[14]^表明，miRNA能直接指导靶mRNA的剪切。植物miRNA大多是调控发育与细胞分化的基因。miR-165/166的靶基因*PHB*与PHV中的序列发生突变后则造成功能获得性突变，*PHB* mRNA表达的组织范围相对于野生型*PHB* mRNA扩大了^[15]^。近来的研究发现，植物中的miRNA不仅可以调控细胞周期及细胞分化，还对环境应答和激素应答有一定作用。miR-167和miR-160调节编码植物激素应答因子基因的表达。在拟南芥中，TIR1蛋白因子通过与E3泛蛋白连接酶SCF复合物的吲哚乙酸结合而参与胚轴分化、横向根系形成等生理过程，而miR-393调控该蛋白的表达^[16]^。

## 3 MiRNA与肿瘤耐药

肿瘤的耐药机制复杂，目前，已知化疗耐药的分子机制包括：药物靶点的改变、药物的改变或失活、DNA损伤修复增强、细胞凋亡减少、药物代谢改变以及药物诱导的细胞核型改变等（图 2）^[17, 18]^。同时，细胞修复和细胞死亡通路等亦与之密切相关。目前研究较多的包括与药物外排相关的能量依赖型转运体，如ABC家族的P170、MRP、BCRP、DNA修复系统如错配修复、细胞凋亡相关分子及谷胱甘肽S转移酶等。这些关键基因发生的一系列遗传或表观遗传上的改变是导致耐药性的主要机制之一。此类改变包括基因的突变（mutation）、缺失（deletion）、扩增（amplification）、转置（translocation）、DNA的异常甲基化以及microRNA的转录后调节等多种方式。其中，有研究揭示miRNA与肿瘤细胞对化疗药物的敏感程度密切相关，miRNA成为肿瘤耐药研究领域一个热点。miR-214可靶向PTEN导致PTEN蛋白低表达，Akt通路过度激活从而促进细胞增殖，致使过表达miR-214的卵巢癌病人对顺铂不敏感^[19]^。SIRT蛋白是依赖于NAD的蛋白去乙酰化酶，通过对P53蛋白去乙酰化起到抗凋亡作用，在PC3细胞中过表达miR-34a可下调SIRT1的mRNA，导致细胞周期停滞、细胞生长受到抑制并增强了PC3对喜树碱的敏感度^[20]^。

**2:**
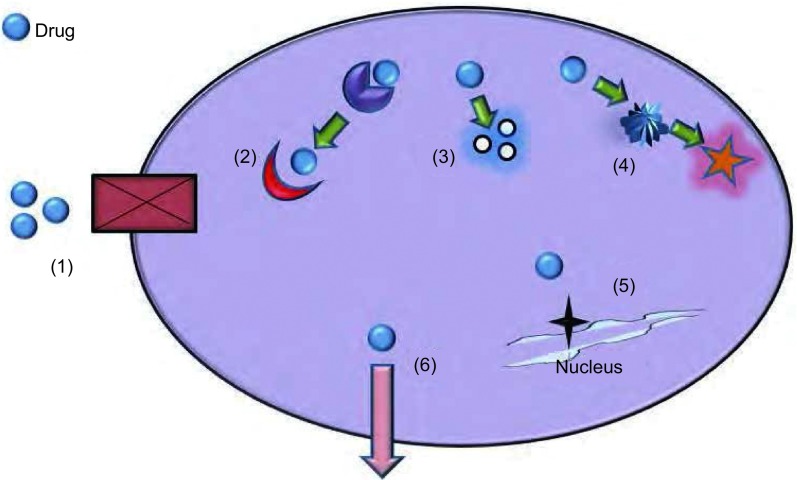
肿瘤细胞耐药的典型分子机制。常见耐药的主要途径有：（1）由于细胞表面受体或转运体缺失导致药物不能进入细胞内；（2）药物靶点改变；（3）药物的改变或失活；（4）药物代谢途径改变；（5）损伤DNA修复增加；（6）药物被主动运输到细胞外。 The molecular mechanisms of drug resistance in tumor cells. The main pathways of drug resistance: (1) The drug can not get into the cell due to lost of cell surface receptors or transporters; (2) Mutations in drug target; (3) Changes or inactivation of the drug; (4) Changes in the drug metabolism pathway; (5) Increased repair of damaged DNA; (6) Drug is exported by active transportation.

miRNA的突变（mutation）、异常表达（mis-expression）和异常加工均会影响miRNA的正常功能，导致靶基因的蛋白表达水平异常。如果此类靶基因与肿瘤细胞对药物的敏感程度相关，将改变肿瘤细胞的药物敏感程度（图 3和表 1）。miR-482-3P靶向抑制NF-Yb转录水平，上调Top2a表达，提高淋巴细胞型白血病CEM细胞对替尼泊苷的敏感性，而下调其表达则促使了CEM/VM-1-5的药物耐受^[21]^。Lu等^[22]^报道胸甘酸合成酶3’UTR区6个碱基的缺失突变与胃癌患者对5-FU的化疗敏感性有关，但具体机制尚不清楚。Tsang的研究^[23]^表明，let-7a通过下调细胞凋亡中的启动酶CASP3抑制了细胞凋亡，在A431和HepG2细胞中过表达let-7a可增强它们对阿霉素、紫杉醇及干扰素的耐药性，抑制let-7a则增加化疗药物引发的细胞凋亡。

**3:**
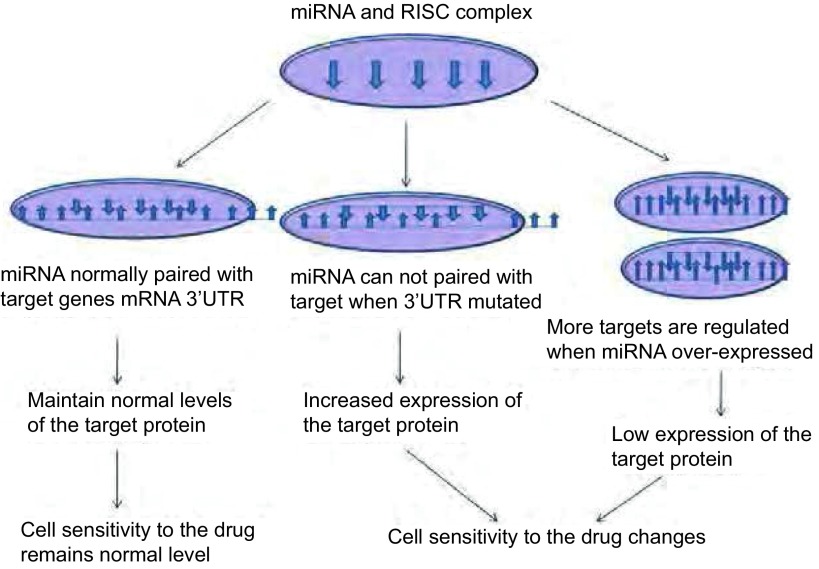
MicroRNA影响药物敏感程度的作用机制。正常情况下，microRNA及靶蛋白的水平维持正常状态；microRNA的基因发生突变时无法与靶mRNA正常配对，靶蛋白高水平表达；microRNA过表达时靶基因低表达。如果此靶蛋白参与细胞对药物的反应，如药物转运体、药物靶点、细胞凋亡及修复等相关蛋白，则导致药物敏感度的改变。 The mechanism of microRNA effect on the sensitivity to drugs. Normally, microRNAs and target proteins are maintain on the normal levels; when microRNA gene mutated, microRNA can not paired with target mRNA, cause the increased expression of target protein; when microRNA overexpressed, the expression of target gene reduced. If the target involves in cell responses to drugs such as drug transporter protein, drug targets, apoptosis or DNA repair related proteins, the sensitivity to drugs will be changed. RISC: RNA-induced silencing complex.

**1:** microRNA在肿瘤细胞耐药中的作用 The role of microRNAs in tumor cells chemoresistance

microRNA	Target genes	Effect	Ref
miR-24	*DHFR*	The single base mutation 829C-T in DHFR 3’UTR, interferes the normal pairing between miR-24 and DHFR’mRNA, which make the expression of dihydrofolate reductase increased, and cause the methotrexate resistant phenotype.	[24]
miR-519c	*BCRP* *ABCG2*	The ABCG2 3’UTR of the S1MI80 cell contained a 1, 500 bp deletion, which includes the miR-519c reactive sites. This mutation make the expression of the drug efflux pump gene: ABCG2 cann’t reduced, and cause the multidrug resistance phenotype.	[25]
Abnormal expression			
miR-15a/16	*BCL2*	The overexpression of miR-15a/16 can reduce the expression of the tamoxifen-induced BCL2, and enhance the resistance of breast cancer cells to tamoxifen.	[26]
		
miR-20	*BNIP2*	miR-20 can modify the sensitivity of colorectal cancer cell lines SW620, SW480 to 5-FU, oxaliplatin and teniposide, overexpression of miR-20 induced SW480 drug-resistance, downexpression of it can increase the drug-sensitivity of SW620.	[27]
miR-21	*BCL2* *MMR* *BCL2* *PTEN* *PI3K*/*Akt*	The overexpression of miR-21 could interfere the colorectal cancer cells G/M arrest and apoptosis induced by 5-FU, that drug-resistant appears by reducing the expression of mutated mismatch repair gene MMR. The overexpression of miR-21 mediates the BCL2/PTEN expression, which caused the DDP-resistance of NSCLC cell. The overexpression of miR-21 can reactivate the expression of tumor suppressor gene PTEN and reduces the activation of PI3K/AKT/mTOR pathway, then increase the sensitivity of pancreatic cancer cells to gemcitabine. Abnormal expression of miR-21 could lead the sensitive response of CML chronic myeloid leukemia cell line K562 to the DNR by changing PTEN expression directly by PI3K/AKT pathway.	[28-32]
miR-27a	*HIPK2*, *MDR1*, *P-gp*	The overexpression of miR-27a which by regulating the target gene of HIPK2, could regulate the expression of MDR1 and P-gp indirectly, which influence ovarian cancer resistant to paclitaxel.	[33]

miR-34	*BCL2* *CCND1* *MAGE-A* *HuR* *SIRT1*	miR-34 suppressed the expression of target genes: BCL2/CCND1, which is leading to the resistance of MCF-7 to docetaxel. The overexpression of miR-34 suppress the expression of MAGE-A, then increasing the expression of P53, and upregulate the expression of miR-34 by positive feedback regulation mechanism, which increases the sensitivity of tumor cells to mitomycin and cisplatin. The overexpression of miR-34 could downregulate the expression of SIRT1 and BCL2 directly or by suppressing the expression of HuR, and weaken the resistance hormone-independent prostate cancer PC3 cells to paclitaxel.	[34-36]
miR-34a	*MYC* *Sirt1*/*E2F3*	The low-expression of miR-34a can increase the expression of myc, and transform the sensitivity of P493-6 cells to brotezomib. Overexpression of miR-34a can antagonist the apoptosis induced by bortezomib. The overexpression of miR-34a can reverse the drug-resistance of DLD-1 cells by suppressing the expression of Sirt1/E2F3.	[37, 38]
miR-199a-3p	Target of rapamycin c-Met	miR-199a-3p can affect the sensitivity of hepatocellular carcinoma cells to doxorubicin by direct role in the target of rapamycin and c-Met, and maintain the low level expression of miR-199a-3p could improve the therapeutic effect of doxorubicin.	[39]
miR-125	*Bak1*	The high-level expression of miR-125 endue the tolerance of breast cancer cells to docetaxel by suppressing the expression of Bak1.	[40]
miR-130b	*CSF-1*	The low expression of miR-130b reduce the efficiency in binding to CSF-1 3’UTR, thereby reducing the sensitivity of ovarian cancer cells to cisplatin and paclitaxel.	[41]
miR-141	*YAP1*	The overexpression of miR-141 can suppress the resistance of esophageal squamous cell carcinoma (ESCC) to cisplatin which is mediated by YAP1 gene.	[42]
miR-143	*KRAS*	miR-143 could increase the drug-sensitivity of prostate cancer by suppressing the expression of target protein KRAS, while the overexpression of miR-143 could also participate the regulation of EGFR/RAS/MAPK pathway and improve the sensitivity of prostate cancer cells to docetaxel.	[43]
miR-146b-3p	*K-ras*	The increase of miR-146b-3p expression could participate the form of in resistance of HCT-116 colon cancer cell to cetuximab.	[44]
miR-193a	p73	The suppression of miR-193a expression caused by inhibiting p-73-mediated feedback regulatory pathway can induce the sensitivity of head and neck squamous cell JHU-029 to chemotherapeutic drugs.	[45]
miR-200c	*PTEN* *Bax*	miR-200c could reverse the resistance of the gastric cancer cells SGC7901 to DDP by upregulating the expression of the target protein PTEN/Bax.	[46]
miR-200bc/429	*Bcl-2* *XIAP*	The enhanced expression of miR-200bc/429 cluster resulting decrease of BCL-2 and XIAP protein, which make the gastric SGC7901/VCR and lung cancer A549/CDDP became sensitive to VCR/CDDP induced apoptosis.	[47]
miR-205/31	*BCL2* *E2F6*	miR-205/31 could suppress the expression of BCL2/E2F6 respectively, and improving the apoptosis of prostate cancer induced by chemotherapy. The downexpression of miR-205/miR-31 plays an important role in the anti-apoptotic function of tumor.	[48]
miR-297	*MRP-2*	In HCT116/L-OHP of the colon cancer cell with multi-drug resistant, the expression of miR-297 was significantly reduced when compared with the parental strain HCT116. Thereby overexpression of miR-297 is expected to improve the drug-resistant of HCT116/LOHP.	[49]
miR-328	*BCRP* *ABCG2*	In MCF-7/MX100 of the drug-resistant cell lines, suppress the expression of miR-328 could promote the expression of BCRP/ABCG2, which could increase drug sinotrans and result the generation of drug resistance.	[50]
miR-451	*MDR1*	In the multi-drug resistant cell lines MCF-7/DOX, overexpression of miR-451 may enhance the sensitivity of cells to doxorubicin.	[51]

## 4 MiRNA在逆转肿瘤耐药中的作用研究

MiRNA究竟通过何种机制对肿瘤细胞的药物敏感程度产生影响尚待研究。2007年，Bertino等^[52]^提出了“miRNA的药物基因组学”（miRNA pharmacogenomics）概念，指出通过研究miRNA及miRNA的突变如何干扰miRNA的正常功能进而改变患者药物反应程度，最终可找出某些特定的miRNA用以指导患者的个性化用药。美国国立癌症研究所对60种癌细胞系（NCI-60）进行了10万多种化学药物的敏感性实验，得到所有细胞系对各种药物的药敏数值GI_50_、LC_50_和IC_50_，同时对这60个细胞系做了DNA、RNA、蛋白质水平的多种分析，包括染色体畸变、DNA拷贝数变化、mRNA及miRNA的表达谱分析，所有数据被整合在CellMiner中，为在分子水平系统性研究肿瘤细胞的药物敏感性提供了数据支持^[53]^。

MiRNA药物基因组学的最终目标是通过分析患者的miRNA及mRNA特征谱，再结合其他分子标志，可以预测患者对某种药物的反应程度，从而因人而异的给予最有效的化学治疗。通过研究miRNA调控肿瘤细胞耐药性，探索miRNA在人类肿瘤治疗中的潜能具有现实意义。*p53*是众所周知的抑癌基因，然而P53相关的转录因子P63/P73在鳞癌中均过表达。一项研究发现化疗可以引发P63/P73依赖性miR-193a-5p的诱导，从而通过miRNA介导的P73的反馈性抑制以限制化疗敏感性。抑制miR-193a可中断该反馈，抑制离体和在体肿瘤细胞的生存并增强化疗敏感性。因此在P53缺失肿瘤中，miR-193a的抑制或可提供新的治疗机会^[54]^。RNAi技术的发展为miRNA调控肿瘤细胞耐药起到了重要意义，如应用反义核酸技术下调miR-21，使miR-21表达减少可以增强乳腺癌MCF-7细胞对拓扑替康的敏感性^[55]^。研究发现miR-200c抑制-微管蛋白3（class Ⅲ-tubulin, TUBB3）表达，过表达miR-200c可恢复子宫内膜癌细胞对化疗药物的敏感性^[56]^。MiR-203过表达通过抑制靶基因*AKT2*的活性，下调其下游耐药相关蛋白MTDH/HSP90的表达，从而增加结肠癌细胞对紫杉醇的敏感性^[57]^。在MDR大肠癌中miR-222与ADAM-17的表达成负相关，提高HCT116/L-OHP和HCT-8/VCR细胞中miR-222的表达量可降低ADAM-17蛋白表达，使细胞凋亡增加，进而改善大肠癌HCT116/L-OHP和HCT-8/VCR细胞对L-OHP/VCR的敏感性^[58]^。肿瘤干细胞（cancer stem cells, CSCs）具有广泛的增殖和自我更新潜能以促进癌症的发展。研究显示let-7和miR-181在肝细胞CSCs中表达上调。还表明let-7靶向作用于SOCS-1和CASP3，而miR-181则靶向作用于RASSF1A、TIMP3及NLK。Let-7的抑制可增加肝细胞CSCs对多柔比星和索拉非尼的化疗敏感性，而miR-181沉默可减弱肝细胞CSCs的活跃性和侵袭性^[59]^。最新研究^[60]^发现，miR-503可能通过抑制药物外排，负调控肿瘤耐药相关蛋白表达，促进细胞凋亡进而逆转肺腺癌A549/DDP细胞对顺铂的耐药性。

## 5 展望

目前miRNA在肿瘤耐药性中的研究尚处于起步阶段，许多具体作用机制还需深入的研究证实，有的研究之间甚至存在矛盾之处。Zhu等^[61]^报道了miRNA-451和miRNA-27a在多药耐药的癌细胞系A2780DX5和KB-V1中表达上调，抑制miR-451或miR-27a的表达可导致MDR1的mRNA和蛋白水平的下降，从而增强细胞系对长春碱、阿霉素的敏感程度。但Kovalchuk等^[62]^研究结果显示，在多药耐药细胞系MCF-7/DOX中过表达miR-451可增强细胞对阿霉素的敏感性。两个研究结果的矛盾说明了miRNA调节机制的复杂性，也表明该领域的不成熟，同样也提醒在研究miRNA时一定要谨慎验证研究结论的可靠性。miRNA表达谱不仅是肿瘤诊断的重要标志物，同时也在肿瘤分期中具有重要作用，近来的研究表明，通过分析药物敏感性及非敏感性细胞的miRNA的表达谱，可以找到影响药物敏感性的相关miRNA，这不仅有利于深入认识肿瘤耐药机制，而且有助于寻找新的药物靶标及为个体化用药提供新的依据。在细胞水平上揭示耐药相关性miRNA及其作用靶点和作用机制只是第一步，这些研究结果最终都要在临床样本中进行验证。

综上所述，miRNA在调节不同肿瘤耐药方面起着重要作用。一个miRNA可调节多个靶基因，一个靶基因也可受多个miRNA的调节，因此miRNA的调节机制是一个复杂的网络结构。正常情况下miRNA通过其精细复杂的调节网络使细胞内各种蛋白的水平维持在平衡状态，miRNA的多态性及表达异常可导致肿瘤耐药相关通路的基因表达水平发生改变、耐药基因和癌基因的高表达、肿瘤细胞周期改变等，从而打破原有的平衡进而改变肿瘤细胞对药物的敏感性，导致耐药现象。MiRNA调控肿瘤细胞耐药是一个庞大而复杂的miRNA-mRNA基因调控网络，因此miRNA完全有潜力成为逆转多因素、多基因导致的肿瘤耐药的高效靶点，从而极大地改善临床肿瘤治疗耐药现状，具有一定的现实意义。相信基于miRNA的分子靶向治疗必将成为临床攻克肿瘤耐药的一种高度特异性的，潜在高效的个体化治疗手段，开启逆转肿瘤耐药的miRNA基因疗法新篇章。
